# GPs’ perspectives regarding suicide prevention: a systematic scoping review

**DOI:** 10.3399/BJGPO.2024.0225

**Published:** 2026-01-14

**Authors:** Jack Marshall, Phillip Oliver, Joe Hulin, Vyv Huddy, Caroline Mitchell

**Affiliations:** 1 Division of Population Health, School of Medicine and Population Health, Regent Court (ScHARR), Sheffield S1, DA, Sheffield, UK; 2 Department of Psychology, Cathedral Court, Sheffield, UK

**Keywords:** mental health, family medicine, suicide, general practice

## Abstract

**Background:**

Suicide is a major public health issue. More than one third of patients will visit their GP in the month leading up to a suicide attempt, thus highlighting the key role GPs play in suicide prevention.

**Aim:**

To explore the qualitative research on GPs’ perspectives of suicide prevention in primary care.

**Design & setting:**

A systematic scoping review of qualitative studies relating to the research question.

**Method:**

This review is reported in accordance with Preferred Reporting Items for Systematic reviews and Meta-Analyses extension for Scoping Reviews (PRISMA-ScR) guidance. Articles at full-text review were assessed for inclusion in the study against eligibility criteria (English language, qualitative research, focus on GPs’ perspectives of suicide prevention). Data were extracted using a standardised form and a thematic synthesis approach was used to describe the themes elicited from the studies.

**Results:**

In total, 2210 abstracts were screened. Twelve studies from seven countries were included at full-text review. The following four main themes were elicited: challenges to managing suicidal behaviour; fragmented relationships with mental health services; personal attitudes of GPs regarding suicidal behaviour; and identified needs to improve suicide prevention in primary care.

**Conclusion:**

Understanding GPs' perspectives can lead to improved training, resources, and support for primary care professionals, who are frontline providers of mental health care. This scoping review suggested there is a lack of evidence around what approaches GPs find effective in managing suicidality and how relationships can be strengthened with mental health services to deliver person-centred integrated care for those identified at risk of suicide.

## How this fits in

Suicide is a major public health issue. GPs face challenges when managing suicidal behaviour in clinical practice. This scoping review summarises these challenges as well as highlighting the need for better service provision from secondary care and further research into what approaches GPs find effective when managing suicidal behaviour.

## Introduction

Suicide is a major public health concern and is the largest cause of death of men aged <50 years in the UK.^1^ While risk factors for suicidal behaviour are known, empirical data and theoretical models highlight the complex nature of suicide, which are not merely explained by the presence or absence of risk and protective factors.^1–4^ This makes it challenging for clinicians to identify and manage these patients.^5–7^ Primary care is the first point of contact for patients with deteriorating metal health in the UK, and 40% of GP appointments involve a mental health complaint.^8,9^ Approximately 40% of patients visit their GP in the month leading up to their suicide attempt.^4,8,10,11^

Suicide prevention involves measures to prevent death by suicide and requires collaboration between health services, local authorities, and third sector suicide prevention organisations.^12^ GPs play a key role by identifying and supporting high-risk individuals by referring patients to mental health services as well as voluntary, community, and social enterprise (VCSE) services to help address risk factors for suicide such as self-harm, substance misuse, and adverse life experiences.

A systematic review and meta-analysis of 14 studies found that interventions targeting GPs produced equivocal results.^11^ This and other reviews have focused primarily on the analysis of quantitative data and there is no current review of qualitative literature pertaining to GP perspectives of suicide prevention.^11,13^ A review of qualitative research enables a greater insight into the personal and professional experiences of GPs, which can be used to inform better resources and training for primary care professionals who are at the frontline when providing mental health care.^14^ The systematic scoping review aimed to explore the qualitative literature looking at GPs’ perspectives of suicide prevention in primary care. One rationale for using a scoping approach is to provide an overview of a potentially large and diverse body of literature pertaining to a broad topic.^15^ This will help to inform key research priorities for further work in suicide prevention.

## Method

This scoping review is reported in accordance with the methodologies of Arksey and O’Malley (2005) and the Preferred Reporting Items for Systematic reviews and Meta-Analyses Extension For Scoping Reviews (PRISMA-ScR).^15–17^ The protocol is registered with an online repository.^18^ A Population, Concept, and Context (PCC) framework was used to develop the research question and search strategy (Table 1).

**Table 1.** Population, Concept, Context framework for developing the research question and search strategy

Population	General practitioner, GP, family doctor
Concept	Suicide prevention, barriers, facilitators, support, qualitative, suicide
Context	Primary care, general practice

### Identifying relevant studies

Studies were identified using a three-step search strategy informed by the Joanna Briggs Institute (JBI) (Supplementary material 1).^16^ An initial search of three online databases (PubMed, Web of Science, and APAPsych info) was carried out using words informed from the PCC framework. These databases were chosen as they are relevant to healthcare and mental health research. A second search of these databases was carried out using further search strategies obtained from analysis of the titles of the articles yielded from the first search. Finally, the reference list of all articles at full-text review were examined for further studies for inclusion in the review.^15,16^ The searches took place between 8 August 2023 and 8 September 2023.

### Study selection

Study eligibility was determined by an initial screen of journal article abstracts against our inclusion criteria. The same criteria were applied at full-text review. Only journal articles were included if they were (i) English language, (ii) had a focus on the perspectives of GPs regarding suicide prevention, and (iii) reported qualitative data either alone or as part of a mixed-methods study. There was no restriction on the date of publication nor country of origin of the research.

### Data extraction and reporting the results

One researcher (JM) performed data extraction using a standardised form and critically appraised the studies using a Critical Appraisal Skills Programme (CASP) tool (Supplementary Tables 1&2).^15,16,19^ Two researchers (JM, CM) independently used a thematic synthesis to describe the main themes identified from included studies.^20^ The researchers independently coded the findings of the included studies to produce descriptive themes.^20^ The researchers then discussed these findings to produce analytical themes that address the research question.^20^

## Results

### Selection of sources of evidence

**Figure 1.**
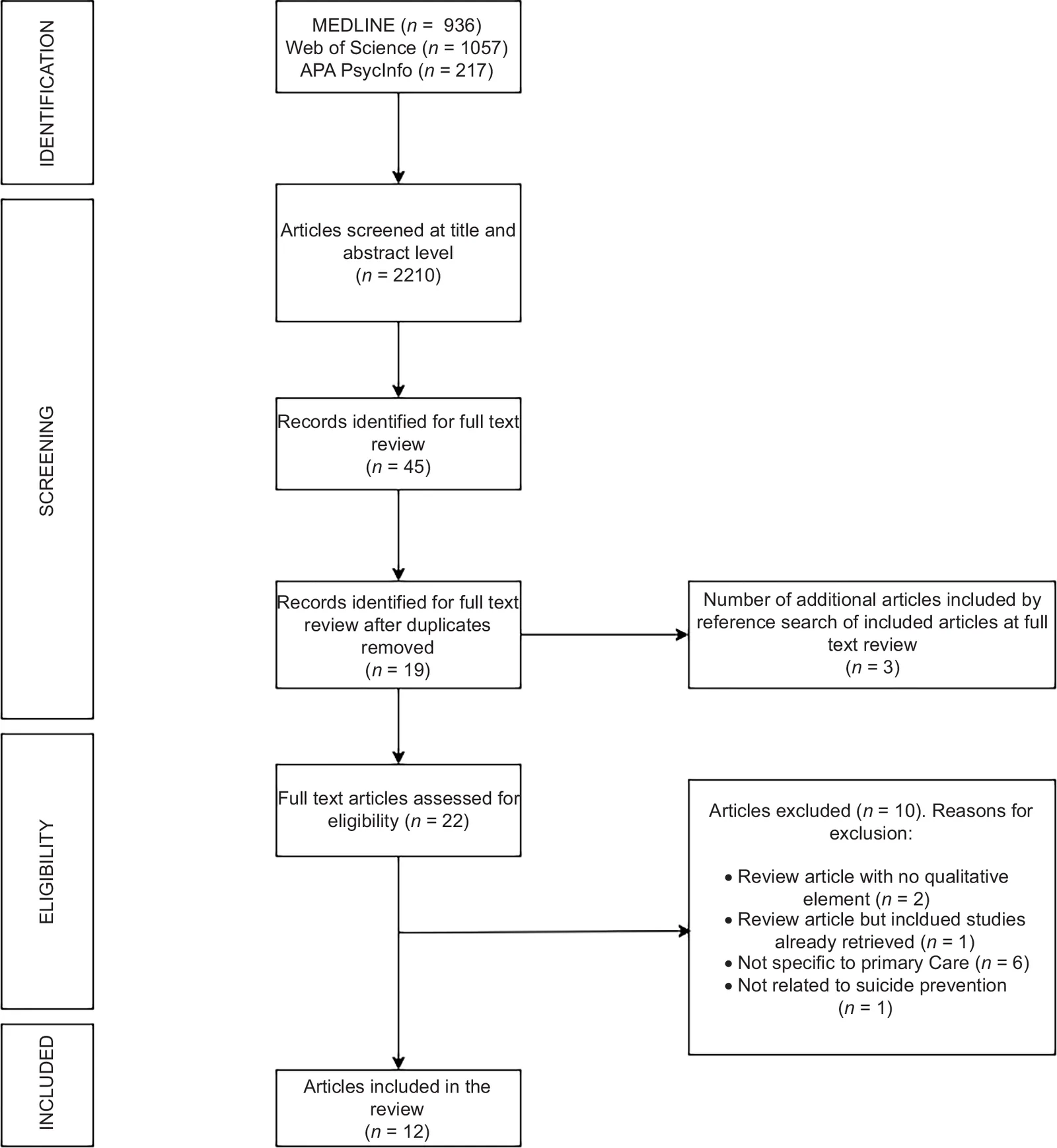
Preferred Reporting Items for Systematic reviews and Meta-Analyses (PRISMA) flow diagram showing study selection

The study selection process is displayed in Figure 1. A total of 2210 abstracts were screened for inclusion. After removing duplicate studies, 19 articles underwent full-text review against the eligibility criteria and 10 articles were excluded. Three articles were retrieved after performing a reference search of included studies at full-text review. A total of 12 articles were included for analysis.^7,21–31^

### Characteristics of sources of evidence

Twelve studies conducted between 2010 and 2022 from seven countries were included (Supplementary Table 3). A total of 353 GPs were interviewed across these studies. The majority of studies originated from the UK, most recently in 2017.^7,22,24,26,27,29^ The other studies originated from Australia (*n* = 1), the Netherlands (*n* = 1), Nicaragua (*n* = 1), Uganda (*n* = 1), Spain (*n* = 1), and Norway (*n* = 1).^21,23,25,28,30,31^

### Synthesis of results

Four main themes relating to GPs’ perspectives on suicide prevention were identified (Table 2). Supplementary Table 3 provides further details on the study characteristics and supplementary Table 4 provides further information relating to data extraction and coding.

**Table 2.** Themes and sub-themes elicited from the included studies

Theme	Sub-theme	Quote
Challenges to managing suicidal behaviour	Challenges of the patient population	*‘It’s more difficult to, I suppose, gauge the risk . . .* [for] *teenagers it’s such a difficult time, and there’s a lot of emotional upheaval.’^21^*
	Time constraints	*‘We are not trained enough ... we cannot go deeper ... For instance, someone may say they wanted to kill themselves because their husband left them, and you cannot understand why because you have no time to go deeper and lack knowledge on what to ask.’^28^*
Fragmented relationships with mental health services	Lack of support from secondary care	*‘Waiting times and a lack of beds is a problem. Sometimes patients who are referred for assessment cannot be admitted as there are no beds. Sometimes patients have to wait too long.’^26^*
	Poor communication	*‘GPs do not have quick access to support services within mental health services, especially at early stages where they have no immediate access. This may be due to the CMHT not allowing immediate access as they have very rigid criteria. Therefore need faster assessments for vulnerable patients, especially if the GP has assessed them and thinks they are in need of some treatment.’^26^*
Personal attitudes of GPs regarding suicidal behaviour	Uncertainty and complexity in suicide risk assessment	*‘It’s very difficult to find who’s really suicidal because, as I* *said, we do, most of the time, they will not come here and say I’m suicidal.’^7^*
	GPs’ perceptions of the relationship between self-harm and suicide	*‘In my experience it seems like the majority of self-harmers didn’t seem to have that high a risk of completing a suicide. In my experience most of them are fairly low risk* [...] *A lot of them were cry for helps.’* ^22^
	The use of screening tools	*‘So in terms of assessment, I don’t use a risk assessment tool or anything, and I kind of weigh what they’re actually saying, in terms of what they’re planning and what’s their history, so I guess I do take that into consideration, and their social situation as well.’^22^*
Identified needs to improve suicide prevention in primary care	Wider community-based support	*’As a health centre we need to re-activate the teen club with the idea of encouraging activities for young people, sports, music, meetings between the clubs, so that will help them to do other things, like having their minds occupied also at school, giving them small workshops or inviting them to come for the different workshops and activities.’^25^*
	Working with primary care mental health support staff	*‘I think you should make people aware that MHSS play an important role in this; they may even be more important than GPs*. *They often have more time*, *expertise*, *and experience than GPs in dealing with these issues*. [This helps] *especially when you are kind of insecure as a GP*.’^23^ *‘I think it’s about time that people turn around and say maybe we should have CPN*[s] *within the surgeries, and they should have always been in the surgeries. The reason why GPs want CPNs in the surgery is because they want support not only for patients but also for themselves. At the end of the day, I am not a psychiatrist, I’m just a GP and I see things and try to do the best I can.’* ^29^

CMHT = community mental health team. CPNs = community psychiatric nurses. MHSS = mental health support staff

#### Challenges to managing suicidal behaviour

GPs described different challenges when managing suicidal behaviour. Particular challenges in risk assessment were described according to different patient populations and GPs across all studies described time constraints within a consultation as a barrier to managing suicidal behaviour. Opinions relating to the use of screening tools to assess risk were generally negative across the included studies.

##### Challenges of the patient population

Three studies focused on suicidal behaviour in teenagers and young adults.^7,21,25^ GPs found risk assessment challenging in this population when trying to differentiate between a mental health problem and emotional dysregulation.^21^

*‘It’s more difficult to, I suppose, gauge the risk . . .* [for] *teenagers it’s such a difficult time, and there’s a lot of emotional upheaval.’^21^*

GPs felt the presence of parents or family members can complicate risk assessment in this population. ^7,21,25^ The appointment may have been made by the caregiver, meaning the patient may be reluctant to share their thoughts.^21^

*‘Sometimes they’ve been brought in by their parents and the parent talks for them, and sometimes* [the] *patient doesn’t even want to be here.’ ^21^*

Patients may also feel unable to discuss their mental health if it is being impacted by family circumstances.^7,21^

*‘... people, there’s something, you know, more deep-rooted or they don’t want to talk against, if there’s something in the family, if they’ve come in with the family, that sometimes can be a bit difficult …’^7^*

Chandler *et al* 2016 noted that preventing suicide can be particularly challenging in populations experiencing health inequalities.^22^ GPs described this population as voicing a ‘*gross ambivalence*’ about being alive, which may be related to their life circumstances.^22^ These patients frequently mention suicidal thoughts within consultations, which can be unclear to the clinician as to whether this is indicative of active suicidal intent or a way of voicing frustrations at their life circumstances.^22^

*‘I think many of them have a wish not to be there. You know, they have passive suicidal ideation; they just wish they didn’t exist anymore.’^22^*

##### Time constraints in the consultation

Short consultation times were frequently mentioned as a challenge as GPs found it difficult to build a rapport with patients and address all the underlying factors that might be contributing to increasing suicidality.^21,23,25,30^

*‘... we cannot go deeper ... For instance, someone may say they wanted to kill themselves because their husband left them, and you cannot understand why because you have no time to go deeper and lack knowledge on what to ask.’^28^*

Rapport-building takes time, especially in the context of managing mental health conditions.^21,25,30^ A good rapport allows the patient to build trust towards their healthcare professional and makes them more likely to engage with treatment plans and follow-up appointments, which are vital in the context of managing suicidal thoughts.^32^

### Fragmented relationships with mental health services

GPs described challenging relationships with mental health services. In particular, clinicians voiced a lack of support in managing acutely unwell patients and raised concerns about how difficult it can be to refer patients into community mental health teams.

#### Lack of support from mental health services

GPs often felt frustrated by a lack of secondary care services.^7,23,26^ Saini *et al* (2010) reported that GPs were concerned about the lack of available beds for those patients needing an admission as well as the rigid referral criteria put in place by their local community mental health team.^26^

*‘Waiting times and a lack of beds is a problem. Sometimes patients who are referred for assessment cannot be admitted as there are no beds. Sometimes patients have to wait too long.’^26^*

Michail and Tait (2016) reported that GPs have to ‘*force*’ crisis teams to assess patients they deemed high risk.^7^ This has led to GPs feeling isolated and taking more responsibility for managing acutely unwell patients.^21,29^

#### Poor communication

Elzinga *et al* (2020) reported that GPs described poor communication of secondary care discharge plans to the community teams and described patients as being ‘*bounced around*’ mental health services.^23^ This is particularly pertinent as individuals recently discharged from mental health services are at higher risk for dying by suicide.^33^

*‘If you send patients to MHC, I sometimes say, “They disappear into a black box. That box is shaken a couple of times and at some point, they fall out.” I never know what happens to my patients in the meantime ...’^23^*

### Personal attitudes of GPs regarding suicidal behaviour

GPs described their personal views towards the challenges associated with assessing suicidal risk and the use of screening tools. GPs in the included studies were divided about the relationship between self-harm and suicidality.

#### Uncertainty and complexity in suicide risk assessment

A common theme was that GPs find it difficult to predict suicide as the presentation of suicidal behaviour can be subtle and difficult to detect unlike a physical health complaint.^7^

*‘It’s more difficult, though, isn’t it, the psychological problems rather than physical where you say temperature is, blood pressure is ... pulse is ...’^7^*

GPs voiced disbelief concerning patients who they deemed to be low risk after an assessment to then go on and die by suicide.^27^

*‘She said, “Doctor, everything is fine ... and then, two days later, the police walked in; she had been on the booze and hanged herself. What do you do about that?”’ ^27^*

GPs also described patients feigning suicidal behaviour owing to ulterior motives when speaking with their doctor, thus making risk assessment even more challenging.^27^

*‘A lot of people sick, mentally ill, and a lot of people pretending to be mentally ill to get disability living allowance.’* ^27^

#### The use of screening tools

Consistent with UK national guidelines, which cautions against the use of risk assessment tools, GPs often stated that they would not use such screening tools for suicide risk assessment. GPs prefer to personalise and contextualise assessment of suicide risk within standard GP consultation frameworks for person-centred care.^22,27^

*‘So in terms of assessment, I don’t use a risk assessment tool or anything, and I kind of weigh what they’re actually saying, in terms of what they’re planning and what’s their history …’ ^22^*

#### GPs’ perceptions of the relationship between self-harm and suicide

Chandler *et al* (2016) looked at the views of GPs relating to self-harm and suicide.^22^ Some GPs in this work described self-harm and suicide as separate entities and that a patient self-harming may not itself be an indicator of suicide risk.^22^

*‘In my experience it seems like the majority of self-harmers didn’t seem to have that high a risk of completing a suicide. In my experience most of them are fairly low risk* [...] *A lot of them were cry for helps.’^22^*

In the same study, other GPs described that self-harm and suicidal behaviour are related and that performing a risk assessment in this population is more challenging as it is difficult to distinguish whether the increase in self-harm behaviour is a means of coping with their mental health, or an indicator of increasing suicidal risk.^22^ GPs interviewed by Saini *et al* (2016) reported self-harm as *‘attention seeking*’ and that ‘*people who talk about suicide don’t do it*’.^29^ The idea of self-harm as a means of ‘*attention seeking*’ was similarly reported by GPs in Michail and Tait in the context of young adults.^7^

### Identified needs to improve suicide prevention in primary care

GPs described a need for greater support from the mental health team in community settings to manage acutely unwell patients and a need for further training in suicide prevention alongside mental health professionals, especially for certain patient populations such as young adults.

#### Working with primary care mental health support staff

GPs often stated that training in suicide prevention should be done alongside trained mental health professionals and patients with lived experience of self-harm and suicidal behaviour.^7,21–23,29,31^ Obando Medina *et al* (2014) described the need for further training for assessing risk in younger patients.^25^

*‘You need skills to work with these patients — not everyone can work with these kinds of patients ... these are young people who cannot be easily addressed and for that we need someone that can help us.’ ^25^*

Saini *et al* (2016) described frustration from GPs concerning the lack of support from secondary care and welcomed the idea of psychiatric nurses within the community to help manage patients.^29^

*‘I think it’s about time that people turn around and say maybe we should have CPN*[s] *within the surgeries, and they should have always been in the surgeries. The reason why GPs want CPNs in the surgery is because they want support not only for patients but also for themselves ...’ ^29^*

#### Wider community-based support

GPs would prefer a broader service incorporating third sector organisations and community groups to holistically manage all the different factors contributing to a patient displaying suicidal behaviour.^22,24,25^ The positive involvement of third sector organisations has been highlighted in work by Abou Seif *et al* (2022), who demonstrated the importance of these groups providing a sense of community in individuals who self-harm.^34^

## Discussion

### Summary

This scoping review demonstrates the common barriers and challenges GPs face in providing adequate suicide prevention responses, such as patient populations and relationships with secondary care services. GPs feel more specialist training is needed alongside improved relationships with mental health teams to manage patients displaying suicidal behaviour.

### Strengths and limitations

There has been previous quantitative research into suicide prevention in general practice but no previous qualitative scoping review has been identified.^11,35^ Saini *et al* (2024) have published a scoping review looking at how primary care practitioners (PCP) can effectively recognise and respond to suicide risk factors(^36^). Our review focused on GP perspectives regarding suicide prevention as a whole and not just the risk assessment. We therefore believe this is the first narrative synthesis of international qualitative research on this topic. A rigorous search strategy of international literature was used including a reference search of studies included at full-text review. Owing to resource constraints, the study searches and data extraction were carried out by one researcher (JM), which may lead to bias from that researcher’s own experiences and opinions. To mitigate this limitation, two researchers (JM and CM) independently identified emergent themes. These were then discussed and refined within our mental health research group, all of whom have experience in synthesising qualitative data (JM, PO, CM, JH, VH). The PCC framework does not include aspects such as needs, perspectives, and views, which may have yielded additional manuscripts and provided a more comprehensive representation of the literature relating to our research question.

### Comparison with existing literature

There was a consensus around certain key themes across seven countries spanning four continents. In particular, the challenges in accessing timely specialist care for patients expressing suicidal thoughts, who GPs considered ‘high risk’, occurred across most healthcare settings. This contrasts with the access afforded to GPs when referring patients for urgent care for acute physical health conditions.^37^ The issue of ‘parity of esteem’ and inequalities between physical and mental health care has been described on a global scale by the World Health Organization.^38^ Common challenges to implementing suicide prevention in primary care include time constraints and assessing differential suicide risk in different patient populations. This aligns with Bajaj *et al* (2008) who conducted a survey of GPs in North London regarding their views of screening for suicidal ideation.^39^ The two most frequently reported barriers to assessing risk were time constraints and asking sensitive questions relating to suicidal thoughts, via interpreters, to patients from ethnic minorities.^39^ Osborne *et al* (2023) conducted a systematic review looking at the challenges GPs face in addressing suicide risk with young adults.^40^ In this work, GPs frequently reported a need for further training in managing suicidal behaviour in younger adults, which aligns with the views of GPs in this study.^40^ Bajaj *et al* (2008) reported less than half of the study population had received formal training in screening for suicide ideation.^39^ This aligns with a frequently reported theme in this study, with GPs asking for better primary care focused training in managing suicidal behaviour.

Up to half of individuals who die by suicide have a history of self-harm.^41^ Mughal *et al* (2020) conducted a systematic review exploring the role of GPs in the management of self-harm, the results of which are comparable with those in this review.^35^ Mughal *et al* (2020) reported that GPs need more training in managing self-harm (particularly in young adults) and better relationships with mental health services.^35^ A common belief reported by GPs in this work was that self-harm is not linked to suicide and described as ‘*attention seeking*’, despite a history of self-harm being the strongest risk factor for death by suicide.^29,41^ Any training on suicide prevention in primary care should involve how to manage self-harm and perform a psychosocial risk assessment as per recent National Institute for Health and Care Excellence’s (NICE) guidance (2022).^35,42^

### Implications for research and practice

The results of this scoping review show that there is an international consensus to obtain a better understanding of primary care professionals’ perspectives relating to suicide prevention, which can lead to improved training, resources, and support for these professionals, ultimately enhancing their effectiveness in suicide prevention as frontline providers of mental health care. This scoping review revealed a limited number of relevant qualitative studies and none in the UK since 2017. Since 2017, general practice in the UK has experienced significant workforce and planning changes such as the ‘NHS Mental Health Implementation Plan 2019/20–2023/24’ and the expansion of traditional GP roles via the Additional Roles Reimbursement Scheme.^43,44^ Further work is needed to describe what approaches GPs, and other primary care mental health practitioners, currently think are effective in managing suicidal behaviour, particularly in younger patients, who GPs find more difficult to manage, which was not UK specific.^7,21,25^ This is aligns with the recent NICE guidance (2022), with one of the five areas of recommended research being models of care for younger patients.^42^

## Data

The dataset relied on in this article is available from the corresponding author on reasonable request.
